# Evaluation of the Anti-Inflammatory Activity of Raisins (*Vitis vinifera* L.) in Human Gastric Epithelial Cells: A Comparative Study

**DOI:** 10.3390/ijms17071156

**Published:** 2016-07-19

**Authors:** Chiara Di Lorenzo, Enrico Sangiovanni, Marco Fumagalli, Elisa Colombo, Gianfranco Frigerio, Francesca Colombo, Luis Peres de Sousa, Ahmet Altindişli, Patrizia Restani, Mario Dell’Agli

**Affiliations:** 1 Dipartimento di Scienze Farmacologiche e Biomolecolari, Università degli Studi di Milano, Milan 20133, Italy; chiara.dilorenzo@unimi.it (C.D.L.); enrico.sangiovanni@unimi.it (E.S.); marco.fumagalli3@unimi.it (M.F.); elisa.colombo1@unimi.it (E.C.); gianfranco.frigerio@unimi.it (G.F.); francesca.colombo1@unimi.it (F.C.); patrizia.restani@unimi.it (P.R.); 2 Instituto Politecnico de Beja, Beja 7800-295, Portugal; luis.peres@ipbeja.pt; 3 Fakulty of Agriculture, Ege University, Bornova, Izmir 35100, Turkey; altindisli@yahoo.com

**Keywords:** raisins, gastric inflammation, HPLC-DAD, polyphenols, NF-κB, IL-8

## Abstract

Raisins (*Vitis vinifera* L.) are dried grapes largely consumed as important source of nutrients and polyphenols. Several studies report health benefits of raisins, including anti-inflammatory and antioxidant properties, whereas the anti-inflammatory activity at gastric level of the hydro-alcoholic extracts, which are mostly used for food supplements preparation, was not reported until now. The aim of this study was to compare the anti-inflammatory activity of five raisin extracts focusing on Interleukin (IL)-8 and Nuclear Factor (NF)-κB pathway. Raisin extracts were characterized by High Performance Liquid Chromatography-Diode Array Detector (HPLC-DAD) analysis and screened for their ability to inhibit Tumor necrosis factor (TNF)α-induced IL-8 release and promoter activity in human gastric epithelial cells. Turkish variety significantly inhibited TNFα-induced IL-8 release, and the effect was due to the impairment of the corresponding promoter activity. Macroscopic evaluation showed the presence of seeds, absent in the other varieties; thus, hydro-alcoholic extracts from fruits and seeds were individually tested on IL-8 and NF-κB pathway. Seed extract inhibited IL-8 and NF-κB pathway, showing higher potency with respect to the fruit. Although the main effect was due to the presence of seeds, the fruit showed significant activity as well. Our data suggest that consumption of selected varieties of raisins could confer a beneficial effect against gastric inflammatory diseases.

## 1. Introduction

Raisins are dried grapes obtained from different cultivars or varieties of *Vitis vinifera* L. and widely consumed in the Mediterranean area. About 95% of raisins are dried “Thomson seedless” (named also “sultanina”) grapes, followed by the Fiesta (3%) and the “Zante currant” (1.5%) grapes [1]. Raisins have been consumed since around 1400 Before Christ (BC) because of their nutritional value [2]. They are an important source of nutrients such as potassium, magnesium, boron, sugars, soluble (fructo-oligosaccharides and inulin) and insoluble fibers [3]. In addition to the nutritional composition, raisins are rich sources of a wide variety of polyphenols that are considered particularly interesting for their beneficial properties in human health. Among them, the most abundant are flavonols (quercetin and kaempferol derivatives) and phenolic acids (mainly caftaric and coutaric acid). Most of the phenol compounds present in raisins derive from the fresh grapes, but other compounds increase during processing, such as caffeoyl tartaric acid and some quercetin and kaempferol derivatives [4].

In the literature, different studies have illustrated the potential health benefits of raisins, which have been shown to possess a low to moderate glycemic index and a low insulinemic index [5]; moreover, raisins increase the feeling of satiety and decrease food intake [6]. Reduction of low-density lipoprotein (LDL) cholesterol, triglycerides, oxidized LDL and oxidative stress suggests a potential protective effect of raisins, reducing risk factors for cardiovascular disease [7,8]. Even though raisins have a long-standing reputation of a food promoting dental caries, due to the presence of significant amount of sugars, new findings have shown that raisins consumption as such does not drop oral pH below the threshold that contributes to the enamel dissolution; moreover, raisins do not remain on the teeth longer than other foods and inhibit some among the bacteria responsible for dental caries [9].

Despite the high number of studies investigating the biological activity of raisins, only few of them considered the beneficial effect in gastric diseases, a condition widely diffused all over the world and whose prevalence has increased in the last few years [10]. Gastritis is an inflammation of gastric mucosa frequently caused by the presence of the bacterium *Helicobacter pylori* (*H. pylori*). During *H. pylori* infection, gastric epithelial cells show higher levels of cytokines including IL-1β, TNF-α, and IL-8, a potent chemokine playing a key role in gastric diseases. Moreover, IL-8 is the main cytokine released by gastric epithelial cells during gastric inflammation. This response is highly dependent on the NF-κB activation, a transcription factor crucial in gastro-intestinal inflammatory diseases [11]. NF-κB is deeply involved in the control of the transcription of several pro-inflammatory mediators, thus leading to the worsening of inflammatory conditions [12]. Moreover, activation of the NF-κB pathway in gastric epithelial cells has been suggested to play a critical role in *H. pylori*-induced chronic inflammation and gastric carcinogenesis [13].

The search for new compounds able to interfere with these mechanisms by preventing a prolonged inflammation could be useful for human health. Polyphenols are ingredients of botanicals widely consumed all over the world for health purposes, with increased usage in the general population, in many different types of products, including foods and plant food supplements. Methanol extracts from different varieties of raisin have been previously investigated as inhibitors of gastric cancer cell growth; raisins suppressed cell proliferation and decreased mRNA levels of Intercellular adhesion molecule (ICAM)-1 in TNFα-stimulated cells [14]. However, in this study, IL-8 release and mRNA levels were not significantly affected by treatment with high concentrations (500 μg/mL) of raisin extracts.

The aim of this study was to compare the anti-inflammatory activity of the hydro-alcoholic extracts from different varieties of raisins at gastric level, focusing on IL-8 and NF-κB pathway. The choice of using a mixture water/ethanol was due to the occurrence of these extracts as main ingredients of plant food supplements, according to their safety. Surprisingly, we found that, among the five raisins subjected to the biological activity, only the Turkish variety significantly inhibited IL-8 secretion. Seeds mainly retained the effect, although fruit extract was active as well. Molecular investigations revealed that both fruit and seeds showed inhibition of IL-8 release via different mechanisms.

## 2. Results

### 2.1. Total Phenol Content Assay

The total phenol content in raisin samples ranged between 2.26 ± 0.12 and 147.78 ± 3.32 mg Gallic acid equivalents (GAE)/g raisins (mean ± s.d.). As expected, the highest content was found in raisin seeds from Turkey sample (Turkish raisin extract TRE seeds, 147.78 ± 3.32 mg/g), followed by TRE (15.04 ± 0.09 mg/g), TRE fruits (5.88 ± 0.78 mg/g), and commercial samples commercial raisin extract CRE1 (3.94 ± 0.12 mg/g), CRE2 (3.77 ± 0.12 mg/g), and CRE3 (3.72 ± 0.03 mg/g); the lowest amount of phenols was detected in Portuguese raisin extract PRE (2.26 ± 0.12 mg/g) (Table 1). Our results are in agreement with the literature data [1,15] except for TRE sample, where the total phenol content was 3–6-fold higher than the other samples. This was due to the presence of seeds, according to [16]. Results were compared to data obtained by HPLC-DAD analysis.

### 2.2. Validation of the HPLC-DAD Method

The HPLC-DAD method developed to quantify the main catechins and procyanidins (catechin, epicatechin, epicatechin-3-gallate, and procyanidin B1, B2, B3, and C1) was validated according to the Food and Drug Administration (FDA) Guidelines on Bioanalytical Method Validation [17] (Table 2). Validation parameters of the other phenolic compounds have been reported previously [18].

The results obtained show that the method is precise (RSD% < 15%), sensitive and capable of separating satisfactorily the compounds occurring in raisin extracts.

Figures S1 and S2 show the chromatograms of standard mixtures of flavonols (at 10 μg/mL, detected at 360 nm) and flavan-3-ols and caftaric acid (at 5 μg/mL, detected at 280 nm), respectively.

### 2.3. HPLC-DAD Characterization of Raisin Extracts

Table 3 shows the content (μg/g raisins) of organic acids, flavonols and flavan-3-ols (monomers, dimers and trimers) in raisin extracts. Representative chromatograms of PRE, TRE, TRE without seeds and TRE seeds are reported in Figures S3–S6.

Caftaric acid, quercetin-3-*O*-glucoside, and kaempferol-3-*O*-glucoside were the most abundant phenols present in the extracts; in particular, caftaric acid ranged between 24.92 ± 1.50 μg/g (CRE1) and 74.93 ± 2.27 μg/g (CRE2) (mean ± s.d.). These results are partially consistent with previous studies, which reported higher levels of some compounds (e.g., phenolic acids and flavonols) in raisins compared to fresh grapes; this is due to the removal of water during processing and to both enzymatic oxidation and non-enzymatic browning reactions occurring during dehydration of grapes [1]. It is noteworthy that catechin, epicatechin, procyanidin B1 and procyanidin B3 were the most abundant compounds in TRE seeds. It is reported that procyanidins, originally present in grapes, appear to be lost during raisins processing [1]. However, since procyanidins can be generally found in the seeds, TRE and TRE from seeds were the only samples that showed high levels of these compounds, in agreement with the literature [19].

### 2.4. Effect of Hydro-Alcoholic Extracts from Different Raisins on IL-8 Release in Human Gastric Epithelial (AGS) Cells

**Figure 1:**
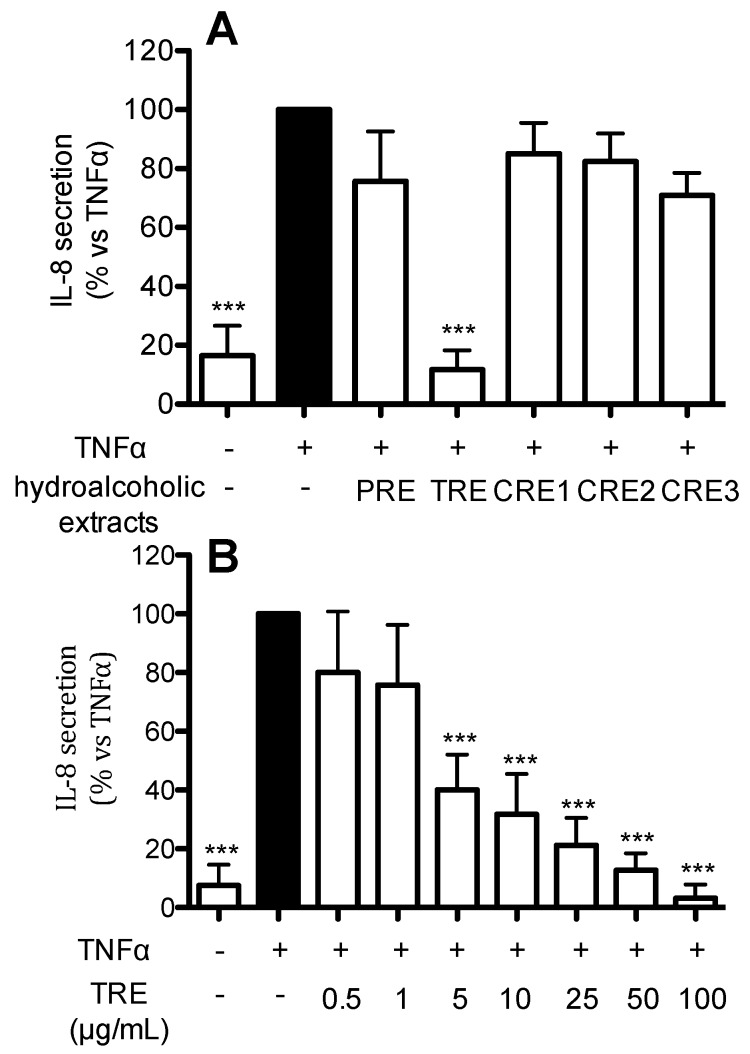
Effect of the hydroalcoholic extracts from different raisin samples on IL-8 release in TNF-α-treated AGS cells. All the extracts were tested at 100 μg/mL to evaluate the inhibitory effect on TNF-α-induced IL-8 (**A**); AGS cells were treated with increasing concentrations of TRE (0.5–100 μg/mL) as described in the Section 4 (**B**). The graphs show the means ± s.d. of at least three experiments performed in duplicates. Statistical analysis: one-way analysis of variance (ANOVA), followed by Bonferroni as post-hoc test. *** *p* < 0.0001 versus TNFα alone. Epigallocatechin-3-*O*-gallate (EGCG) was used as reference inhibitor of IL-8 release (>70% inhibition at 20 μM).

In order to gain new insights into the beneficial effects of raisins at gastric level, we investigated the ability of the hydro-alcoholic extracts from different raisins to inhibit TNFα-induced IL-8 release. Human gastric epithelial cells were treated for 6 h with TNFα in the presence of 100 μg/mL PRE, TRE, and three raisin samples commercially available in Italian drugstores (CRE1-2-3) (panel A). As shown in Figure 1A, all the extracts except TRE were inactive at 100 µg/mL. Then, the Turkey sample was further investigated in a concentration response experiments (0.5–100 μg/mL); as shown in Figure 1B, TRE was able to inhibit TNFα-induced IL-8 release in a concentration-dependent fashion, showing an IC_50_ of 3.34 µg/mL.

Macroscopic evaluation of the Turkey raisin showed the presence of big seeds absent in the other varieties considered in the present study.

### 2.5. TRE from Fruits Inhibits IL-8 Release and Promoter Activity by a NF-κB Independent Mechanism

**Figure 2:**
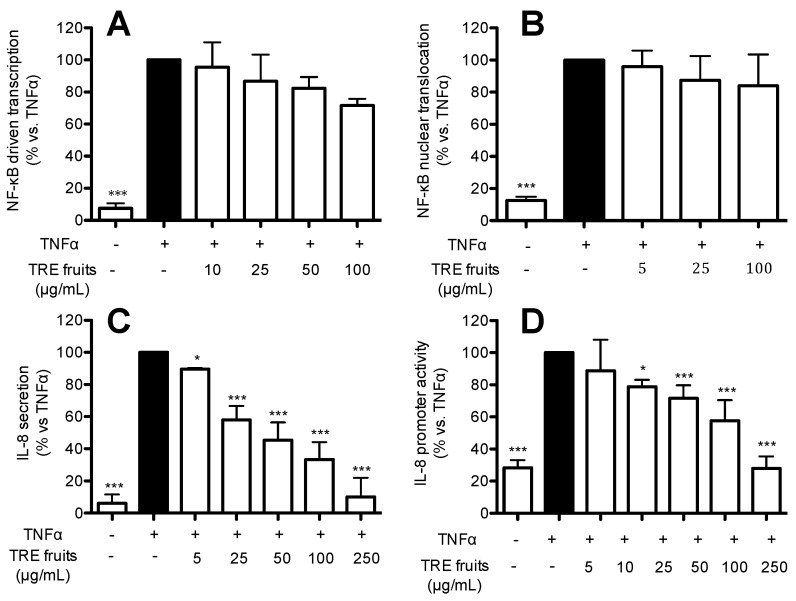
Effect of TRE fruits on NF-κB pathway (**A**,**B** for driven transcription and nuclear translocation, respectively), IL-8 secretion (**C**) and promoter activity (**D**), in TNF-α-treated AGS cells. Graphs show the means ± s.d. of at least three experiments performed in duplicates (ELISA assays) or triplicates (transfections). Statistical analysis: one-way analysis of variance (ANOVA), followed by Bonferroni as post-hoc test. * *p* < 0.05, *** *p* < 0.0001 versus TNFα alone. The treatment with the reference inhibitor (20 μM EGCG) yielded the expected inhibition of the tested parameters: >85% inhibition of NF-κB driven transcription, >90% inhibition of p65 translocation; >70% inhibition of IL-8 secretion; and >80% inhibition of IL-8 promoter activity.

To gain further insights into the portion of the raisin mainly responsible for the biological activity at gastric level, we subjected TRE, obtained from fruits deprived of seeds, to a panel of experiments; since it is widely reported in the literature that IL-8 expression is dependent on the NF-κB activation, contributing to exacerbate inflammation in this district, the following experiments were devoted to evaluate the effect of TRE on the NF-κB pathway and IL-8 secretion and promoter activity induced by TNFα. NF-κB driven transcription was assessed in AGS cells transiently transfected with the NF-κB-LUC plasmid and treated for six hours with TNFα (10 ng/mL) in the presence of increasing concentrations of TRE without seeds (Figure 2A). The amount of p65 translocation was measured by ELISA and normalized by protein content (Figure 2B). The amount of IL-8 released into the medium (Figure 2C) was measured as indicated in the material and methods section, whereas the effect on IL-8 promoter activity was evaluated in AGS cells transiently transfected with IL-8-LUC and treated for six hours with TNFα (10 ng/mL) in the presence of increasing concentrations of TRE without seeds (Figure 2D). As show in Figure 2A,B, TRE from fruits did not significantly affect TNFα-induced NF-κB driven transcription even at high concentrations (100 μg/mL) whereas no effect on nuclear translocation was observed. Conversely, the extract was able to inhibit IL-8 release and promoter activity in a concentration dependent fashion (Figure 2C,D), with an IC_50_ of 37.8 and 39.5 μg/mL, respectively (Table 4). The IC_50_ on IL-8 release highly resembled that obtained on IL-8 promoter activity, thus suggesting that the inhibition of IL-8 release reflects an impairment of the corresponding gene expression.

### 2.6. TRE from Seeds Inhibits IL-8 Release and Promoter Activity by a NF-κB Dependent Mechanism

**Figure 3:**
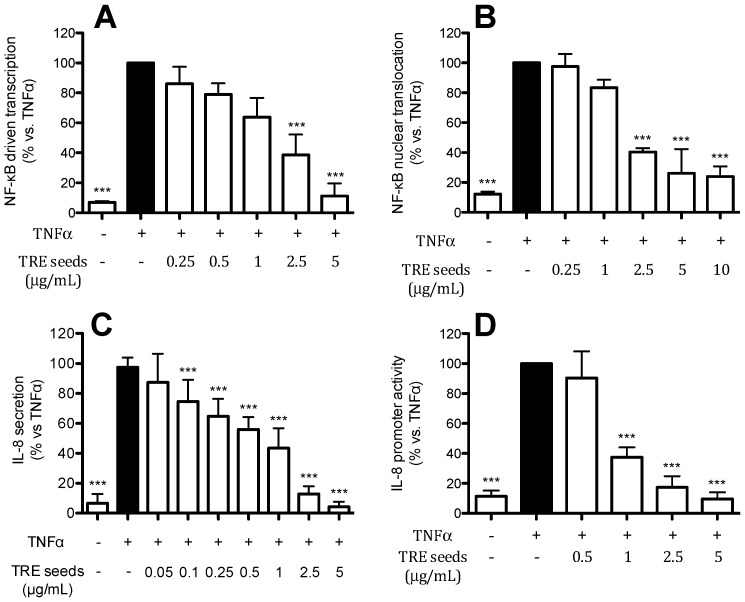
Effect of TRE seeds on NF-κB pathway (**A**,**B** for driven transcription and nuclear translocation, respectively), IL-8 secretion (**C**) and promoter activity (**D**), in TNF-α-treated AGS cells. The graphs show the means ± s.d. of at least three experiments performed in duplicates (ELISA assays) or triplicates (transfections). Statistical analysis: one-way analysis of variance (ANOVA), followed by Bonferroni as post-hoc test. *** *p* < 0.0001 versus TNFα alone. The treatment with the reference compound (20 μM EGCG) yielded the expected inhibition of the tested parameters: >85% inhibition of NF-κB driven transcription, >90% inhibition of p65 translocation; >70% inhibition of IL-8 secretion; and >80% inhibition of IL-8 promoter activity.

To investigate the contribution of seeds to the biological activity observed with TRE, seeds from Turkey raisin were collected and subjected to the hydro-alcoholic extraction as described in the materials and methods section. The extract was then tested on the NF-κB pathway and IL-8 release and promoter activity in human gastric epithelial cells. TRE from seeds was able to inhibit TNFα-induced NF-κB driven transcription and nuclear translocation in a concentration-dependent manner (Figure 3A,B) with very low IC_50_ (1.34 and 1.81 μg/mL, respectively, Table 4). When we examined the effect on IL-8 release and promoter activity in TNFα-treated cells, the extract showed a concentration dependent inhibition with IC_50_ below 1 μg/mL (0.49 and 0.86 μg/mL, respectively). TRE from seeds showed inhibition of IL-8 promoter that resembled the effect on IL-8 release, and the effect was due, at least in part, to the inhibition of NF-κB pathway.

### 2.7. Contribution of Individual Compounds to the Anti-Inflammatory Activity of TRE from Seeds

**Figure 4:**
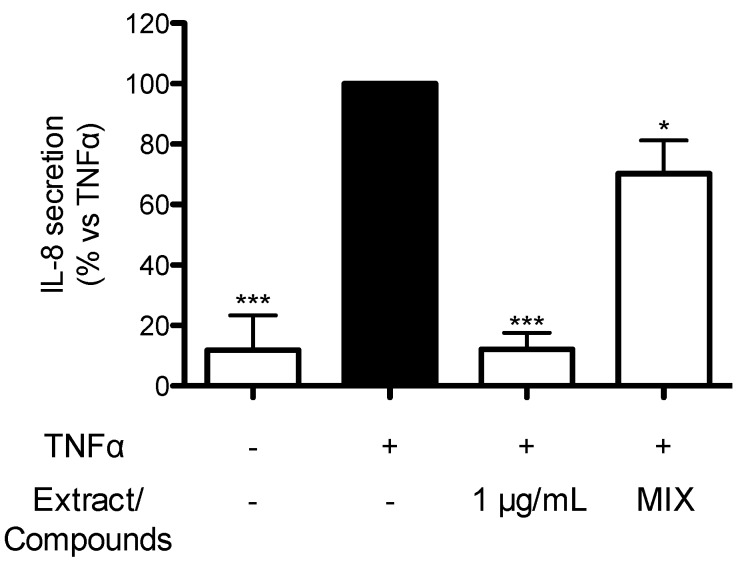
Effect of the mixture of pure compounds on TNFα-induced IL-8 release in AGS cells. Graphs show the means ± s.d. of at least three experiments performed in triplicates. Statistical analysis: one-way analysis of variance (ANOVA), followed by Bonferroni as post-hoc test. * *p* < 0.05, *** *p* < 0.0001 versus TNFα alone. EGCG was used as reference inhibitor of IL-8 release (>70% inhibition at 20 μM).

In order to understand which compounds could contribute to the biological activity of TRE from seeds, the most abundant compounds, measured in the extract by HPLC-DAD, were tested as purified standards. (+) catechin (150 nM), epicatechin (50 nM), epicatechin-3-gallate (2.5 nM), epigallocatechin-3-gallate (0.165 nM), procyanidin B1 (11 nM), procyanidin B2 (4 nM), procyanidin B3 (11 nM), and procyanidin C1 (3 nM) were mixed at concentrations occurring in TRE seeds (1 μg/mL, concentration that completely inhibited TNFα-induced IL-8 release). The mixture was tested on TNFα-induced IL-8 release in comparison to TRE seeds. As shown in Figure 4, the mixture showed 30% inhibition of TNFα-induced IL-8 release, thus suggesting that pure compounds present in the mixture contribute, at least in part, to the anti-inflammatory activity elicited by the extract. However, other compounds, still unknown, are supposed to contribute to the effect.

## 3. Discussion

There is increasing evidence supporting the beneficial effects of botanicals and natural compounds against gastrointestinal inflammation [18,20,21]. Grapes have been grown for thousands years and were dried into raisins as early as 1400 BC [2]. Raisins keep on being an important grape product, consumed for their nutritional value. According to the literature, in the last few years, the interest in their potential health benefits, not only as food but also as a possible ingredient of plant food supplements, has increased. Despite the evidence supporting the benefits in human health, the anti-inflammatory effect of raisins has not fully elucidated [1].

In this study, hydro-alcoholic extracts were prepared from Turkish and Portuguese varieties and commercial samples, in order to possibly identify cultivars with the most promising anti-inflammatory activity at gastric level. For the present study, we have chosen three varieties commercially available in Italian drugstores, and two raisin samples provided by small producers in countries widely devoted to raisin production (Turkey and Portugal).

The first aspect evidenced by this work is a wide variability in terms of qualitative/quantitative phenolic composition and biological activity of tested raisin samples. Caftaric acid and quercetin-3-*O*-glucoside were present in all the varieties assayed, according to the literature data, where is reported that these compounds are not generally affected by processing procedure of raisins [4]. Catechin, epicatechin, procyanidin B1 and procyanidin B3 were present only in TRE seeds and undetectable in TRE fruits. If compared with other samples, quercetin-3-*O*-glucuronide and kaemferol-3-*O*-glucoside were particularly abundant in PRE; this could due to the drying procedure of the raisin that may protect these classes of phenol compounds from oxidation processes. On the other hand, sun-drying and dipping processes could be involved in polyphenol oxidase activation [4]. In addition, also genetic and growth factors are key influencers of a grape cultivar’s phenolic content [22].

In parallel, a significant difference in biological activity was also observed since only Turkish raisin extracts (TRE, TRE fruits and TRE seeds) were able to inhibit, in a concentration-dependent fashion, TNFα-induced IL-8 release, showing an IC_50_ of 3.34, 37.8 and 0.49 μg/mL, respectively. Notably, a mixture containing catechins and procyanidins showed 30% inhibition of IL-8 release thus explaining, at least in part, the effect exerted by the extract obtained from seeds. This observation strongly suggests that other components, still unknown, contribute to the biological activity of the extracts.

The biological effects of grape seeds, mainly associated with procyanidins content, have been widely reported, and their effects as anti-inflammatory agents have been previously published [23,24]. Recently, Adam et al. reported that diabetic rats treated with *Vitis vinifera* L. seed aqueous extract showed lower mRNA levels of the pro-inflammatory mediator tnf-α when compared to non-treated diabetic rats [25]. However, no study regarding the anti-inflammatory activity of raisin seeds has been reported so far. Indeed, even though raisins are produced from *Vitis vinifera* varieties and similar characteristics of the seeds would be expected, technological and drying processes applied to grapes have to be considered, since they could affect their phenolic composition and, consequently, the biological activity. As reported in previous studies, procyanidins and flavan-3-ols are completely degraded in raisins, due to oxidative reactions (non-enzymatic browning, auto-oxidation, oxidation by polyphenol oxidase and peroxidase) occurring during the processing procedures for raisins preparation [22,26]. In our study, only the processing procedures of Turkish and Portuguese raisins were known. The former were sun-dried after dipping processing, while the latter were dried on the plant. Accordingly, degradation processes significantly occurred independently of the drying procedure used. Indeed, apart from TRE and TRE seeds, where the significant contribution of the seeds has to be taken into account, catechin, epicatechin, epicatechin-3-gallate, epigallocatechin-3-gallate and procyanidins were undetectable in all the varieties assayed.

From the mechanistic point of view, the inhibition of IL-8 release by NF-κB dependent mechanism is supported by previous data from the literature, where procyanidins showed an inhibitory activity on NF-κB pathway at different levels, down-regulating I-κB kinase activity, which is the enzyme responsible for the phosphorylation and subsequent degradation of I-κB [27,28] and promoting the retention of NF-κB into the cytoplasm as an inactive complex bound to I-κB. Moreover, procyanidin B2 might regulate NF-κB activation and its interaction with specific DNA binding sites [28,29].

Procyanidins were not detected in TRE fruits, which showed an inhibition of IL-8 release independent from the NF-κB pathway. This observation suggests that other compounds could contribute to the anti-inflammatory activity of the extract acting by different mechanisms, including activator protein (AP)-1. Among the compounds identified in the seeds of Turkish raisin, procyanidins and catechins contribute to the inhibitory effect of the extract on IL-8 release. Literature data show that procyanidins typically occur in grape seeds, and procyanidin B5 and B5-3’-gallate have been showed to possess the highest antioxidant activity in an epidermal lipid peroxidation assay [30]. Unfortunately, these compounds are not commercially available making their quantification difficult.

The Turkish raisin showed promising results as anti-inflammatory agent at the gastric level; this variety could be interesting for a possible use in food market, as an ingredient of plant food supplements or as a food with specific gastric anti-inflammatory effect. In the latter case, one could say that the presence of the seeds is not appreciated by consumers; however, raisins are often used in snacks, mixed up with cereals or other ingredients, so that their perception would be reduced. Noteworthy, the fruit without seeds was active as well.

Considering the whole dried fruit, our results showed that only 2 mg of Turkish variety are enough to carry out a biological activity at the gastric level (IC_50_ of 3.34 μg/mL). Taking into account the weight of a single unit of raisin is 1.81 ± 0.35 g (mean ± standard deviation of 10 of single units of raisin) and the volume of the gastric juice (30–40 mL), is plausible that an intake of raisins as food or supplement might lead to a significant reduction of gastric inflammation.

Taken together, our results suggest that seeds could have an important contribution in the anti-inflammatory activity of raisins, mediated by phenol compounds, and their consumption could confer a beneficial effect to the gastric inflammatory diseases.

## 4. Materials and Methods

### 4.1. Reagents

All reagents used for analytical determinations, (water, acetonitrile, methanol, formic acid and hydrochloric acid) were from VWR International (Fontenay-sous-Bois, France).

Hyperoside (purity > 98%), kaempferol-3-*O*-glucoside (purity > 99%), procyanidin B1 (purity ≥ 80%), procyanidin B2 (purity ≥ 90%), and catechin (purity ≥ 99%) were purchased from Extrasynthese (Genay, France); rutin (purity > 97%), quercetin-3-*O*-glucoside (purity ≥ 98%), quercetin-3-*O*-glucuronide (purity > 98%), epicatechin (purity > 90%), epicatechin-3-gallate (purity > 98%), epigallocatechin-3-gallate (purity > 95%), Gallic acid, sodium carbonate, and Folin–Ciocalteu’s reagent were from Sigma-Aldrich (St. Louis, MO, USA); caftaric acid (purity ≥ 95%) and procyanidin C1 (purity ≥ 80%) were from Phytolab GMBH & Co. (Vestenbergsgreuth, Germany); and procyanidin B3 (purity > 98%) was from Carbosynth Limited (Compton, Berkshire, UK).

### 4.2. Plant Material and Samples Preparation

Five raisins (*Vitis vinifera* L.) were included in the study: Portuguese raisin (variety Early Gold) was a sultanin-type seedless from Beja (Portugal) whereas Turkish raisin (variety sultana) was from Izmir, Turkey. Three brands of raisin (CR-1, CR-2, CR-3) were purchased in Italian drugstores; the origin of CR-1 was not reported in the label, while CR-2 and CR-3 were from Australia and Turkey, respectively. Turkey raisin was prepared as follows: after being harvested, grapes were dipped into a solution for removing wax layer of berries. Solution was composed by water (100 L), potassium carbonate 5% (*w*/*v*) and olive oil 1% (*v*/*v*). Then, grapes were laid on polyethylene sheets and sun-dried for seven days. Raisins were then stored until use. After ripening, Portuguese raisin was allowed to dry on the plant for 5 weeks and then harvested.

A voucher of each raisin commercially available (named CR-01, CR-02, CR-03), from Portugal (named PS-01) and from Turkey (named TS-01) was stored at the Laboratory of Pharmacognosy, Department of Pharmacological and Biomolecular Sciences, Università degli Studi di Milano. Since macroscopic evaluation of variety from Turkey showed the presence of seeds, absent in the other varieties, they were manually removed from a portion of raisin samples. Seeds and fruits obtained after this procedure were analyzed at the same conditions of the other samples.

### 4.3. Preparation of the Hydro-Alcoholic Extract

Hydro-alcoholic extracts from Turkish (TRE) and Portuguese (PRE) raisins were prepared in order to investigate differences in anti-inflammatory activity, and compared to the hydro-alcoholic extracts obtained from raisins commercially available (CRE-1. CRE-2, CRE-3). About 2 g of raisins sample was added to 40 mL of water:ethanol mixture (1:1). Samples were thoroughly homogenized and mixed under stirring for 4 h in dark conditions at room temperature. Then, the mixture was filtered in a vacuum flask and 40 mL of hydro-alcoholic solution were added to the residue and the mixture further incubated overnight at the same conditions described previously. After this procedure, supernatants were combined, freeze-dried and stored at −20 °C until the analysis.

### 4.4. Cell Culture

Human adenocarcinoma cells (AGS) were grown at 37 °C in DMEM F12 (Life Technologies Italia, Monza, Italy) supplemented with 100 units penicillin/mL, 100 mg streptomycin/mL, 2 mM l-glutamine and 10% heat-inactivated fetal calf serum (FCS) (Euroclone S.p.A, Pero, Italy) (complete medium) in a humidified atmosphere containing 5% CO_2_.

### 4.5. Total Phenol Content Assay

Total polyphenol content was determined according to Folin–Ciocalteu’s method, as reported by Singleton and Rossi [31]. Freeze-dried samples (50 mg) were solubilized in 1 mL of a 50:50 water:methanol solution. Aliquots of 300 μL from different samples were mixed in test tubes with 1.5 mL of Folin–Ciocalteu’s reagent diluted 10 times, and 1.2 mL of 7.5% (*w*/*v*) sodium carbonate. After 30 min, the absorbance was measured at 765 nm in a UV-visible spectrophotometer (Varian Cary 50 SCAN, Palo Alto, CA, USA). The polyphenol content in samples was calculated using a standard curve of Gallic acid. Results were expressed as equivalents of Gallic acid in mg/g.

### 4.6. HPLC-DAD Conditions and Method Validation

Chromatographic analysis was conducted according to the method set-up previously [18]. In the present study, validation procedures were also performed for the main flavan-3-ols monomers (catechin, epicatechin, epicatechin-3-*O*-gallate), dimers (procyanidin B1, B2, B3) and trimers (procyanidin C1), according to the FDA Guidelines on Bioanalytical Method Validation [17].

The chromatohraphic column was a Synergi 4u MAX-RP 80A (250 × 2 mm, 4 μm) (Phenomenex, Torrance, CA, USA). The analysis was performed using a gradient elution at a flow rate of 0.3 mL/min, where (A) water:acetonitrile:formic acid 96.9:3:0.1 (*v*/*v*/*v*); and (B) acetonitrile:water:formic acid 50:49.9:0.1 (*v*/*v*/*v*). The gradient was programmed as follows: 0–15 min: 94%–70% A, 15–30 min: 70%–50% A, 30–35 min: 50%–10% A, 35–38 min: 10% A isocratic, 38–48 min: 10%–94% A. The detection was set at 360 nm for flavonols and at 280 nm for caftaric acid and flavan-3-ols.

The HPLC equipment was from Thermo (San Josè, CA, USA) and consisted of a pump (P2000, Thermo Separation Products, San Josè, CA, USA), an interface (SN4000, Thermo Separation Products, San Josè, CA, USA), a Diode Array Detector (6000 LP, Thermo Separation Products) and an injection valve (Rheodyne, Cotati, CA, USA) with a 20 μL loop.

For validation procedures, 1 mg of each standard was diluted in 5 mL of a water:methanol 1:1 solution (*v*/*v*) and thereafter in HCl 0.1 M, in order to obtain the concentrations to be used for linear regression, sensibility and precision tests. Intra-day precision was determined by calculating the Relative Standard Deviation % (RSD%) of the peak areas of five replicates injected in the same day. Inter-day precision was evaluated by repeating the intra-day precision study in three different days.

Calibration curves were prepared by plotting the peak areas of each analyte versus the corresponding concentration and fitted by least-squares linear regression. Each standard solution was analyzed in five separated chromatographic runs. The linearity of the calibration curves was assessed by the correlation coefficient *R*^2^.

For each standard, the limits of detection (LOD) and quantitation (LOQ) were established at a signal-to-noise ratio of 3 and 10, respectively, using ChromeQuest software.

For raisin samples analysis, 50 mg of the lyophilized sample was solubilized in 1 mL of 0.1 M HCl, thoroughly vortexed and centrifuged at 3000 r.c.f. for 10 min. The supernatant was properly diluted and injected into the HPLC. Each analysis was performed at least in triplicate.

### 4.7. Measurement of IL-8 Secretion

Cells were grown in 24-well plates for 48 h (30,000 cells/well). IL-8 secretion (induced by TNFα 10 ng/mL) was evaluated after 6 h treatment in the presence of raisins extract; EGCG (20 μM) was used as the reference inhibitor of IL-8 secretion. IL-8 was quantified using a Human Interleukin-8 ELISA Development Kit (Peprotech Inc., London, UK). Corning 96 well EIA/RIA plates from Sigma-Aldrich (Milan, Italy) were coated with the capture antibody provided in the ELISA kit and incubated overnight at room temperature. The following day, after blocking the nonspecific binding sites in each well, 200 μL of samples in duplicate were transferred into wells at room temperature for 2 h. The amount of IL-8 in the samples was detected by the use of biotinylated and avidin-HRP conjugate antibodies, evaluating the 3,3′,5,5′-tetramethylbenzidine (TMB) substrate reaction. Signal was read using spectroscopy Victor X3 (PerkinElmer, Walthman, MA, USA) at 450 nm 0.1 s. Quantification of IL-8 was done using an optimized standard curve supplied with the ELISA kit (8.0–1000.0 pg/mL). The results are the mean ± s.d. of three experiments in triplicate.

### 4.8. NF-κB Driven Transcription and IL-8 Promoter Activity

To evaluate the NF-κB driven transcription and IL-8 promoter activity, AGS cells were plated in 24-well plates (30,000 cells per well). After 48 h, cells were transiently transfected by the calcium-phosphate method with different reporter plasmids (NF-κB-LUC, 50 ng/well; IL-8-LUC, 100 ng/well); all the plasmids contain luciferase gene under control of a specific promoter: NF-κB-LUC promoter possesses three κB responsive elements, while IL-8-LUC contains a fragment of the native promoter of the human IL-8, gene which is characterized by different responsive sequences for transcription factors such as activator protein 1 (AP-1), CCAAT-enhancer-binding protein-β (C/EBPβ), and NF-κB. The plasmid NF-κB-LUC was a gift of Dr. N. Marx (Department of Internal medicine-Cardiology, University of Ulm, Ulm, Germany) while the plasmid IL-8-LUC was kindly provided by Dr. T. Shimohata (Department of Preventive Environment and Nutrition, University of Tokushima Graduate School, Tokushima, Japan). After 16 h, the cells were treated with the stimulus (TNFα 10 ng/mL) and the extract for 6 h. EGCG (20 μM) was used as the reference inhibitor. At the end of this time, cells were harvested and the luciferase assay was performed using the Britelite™ Plus reagent (PerkinElmer Inc., Walthman, MA, USA) according to the manufacturer’s instructions. Data were expressed considering 100% of the luciferase activity related to the cytokine-induced promoter activity.

### 4.9. NF-κB Nuclear Translocation

To evaluate the effects of the raisins extracts on NF-κB nuclear translocation, human epithelial gastric AGS cells were plated in 100 mm dishes (3 × 10^6^ cells per dish) with fresh medium for 48 h. Then, the cells were treated with different concentrations of extracts in the presence of pro-inflammatory cytokine (TNFα 10 ng/mL) for 1 h. Nuclear extracts were prepared using a Nuclear Extraction Kit from Cayman Chemical Company (Ann Arbor, MI, USA) and stored at −80 °C until assayed. The same amount of total nuclear proteins (10 μg/well), measured by the method of Bradford (Bio-Rad Laboratories, Segrate, Italy), was used to assess the NF-κB nuclear translocation using the NF-κB (p65) transcription factor assay kit (Cayman Chemical Company, Ann Arbor, MI, USA) followed by spectroscopy at 450 nm, 0.1 s (Victor X3, Perkin Elmer, Walthman, MA, USA). Data were expressed considering 100% of the absorbance related to the cytokine-induced NF-κB nuclear translocation. EGCG (20 μM) was used as the reference inhibitor of NF-κB nuclear translocation. The results are the mean ± s.d. of three experiments in triplicate.

### 4.10. Cytotoxicity

The integrity of the cell morphology before and after treatment was assessed by light microscope inspection. Cell viability was measured, after 6 h treatment, by the 3,4,5-dimethylthiazol-2-yl-2-5-diphenyltetrazolium bromide (MTT) method. This method evaluates the activity of a mitochondrial enzyme, which is an index of cell viability. The extracts didn’t show cytotoxicity at each concentrations tested.

### 4.11. Statistical Analysis

All data are expressed as mean ± s.d.; data were analyzed by unpaired one-way analysis of variance (ANOVA) followed by Bonferroni as post-hoc test. Statistical analyses were done using GraphPad Prism 5.0 software (GraphPad Software Inc., San Diego, CA, USA). *p* < 0.05 was considered statistically significant. IC_50_ was calculated using GraphPad Prism 5.00 software.

## Figures and Tables

**Table 1:** Total phenol content of the hydroalcoholic extracts from raisins ^1^.

Measurement	PRE	TRE	TRE without Seeds	TRE Seeds	CRE1	CRE2	CRE3
Total phenol content	2.26 ± 0.12	15.04 ± 0.09	5.88 ± 0.78	147.78 ± 3.32	3.94 ± 0.12	3.77 ± 0.12	3.72 ± 0.03

^1^ Results, expressed as mg gallic acid (GA) equivalents/g raisin, are the mean ± standard deviation (s.d.) of three experiments performed in triplicate. TRE: Turkish raisin extract; PRE: Portuguese raisin extract; CRE: commercial raisin extract.

**Table 2:** HPLC-DAD validation parameters for flavan-3-ols quantification.

Compound	Precision		Linearity		Sensibility	
	Intraday (CV %) ^1^	Interday (CV %) ^1^	Linear Range (μg/mL)	Correlation Coefficient (*R*^2^) ^1^	LOD ^1^ (ng/mL)	LOQ ^1^ (ng/mL)
Catechin	2.52	5.51	0.03–5	0.997	5.0 ± 0.6	16.6 ± 2.0
Epicatechin	3.44	3.90	0.03–5	0.999	4.2 ± 0.5	13.9 ± 1.8
Epicatechin-3-gallate	10.84	2.41	0.03–5	0.991	1.2 ± 0.1	3.9 ± 0.2
Epigallocatechin-3-gallate	3.87	6.63	0.1–5	0.999	2.1 ± 0.1	7.1 ± 0.3
Procyanidin B1	2.29	4.10	0.1–5	0.998	6.9 ± 0.8	22.9 ± 2.8
Procyanidin B2	2.16	4.19	0.1–5	0.995	5.2 ± 0.6	17.4 ± 2.1
Procyanidin B3	3.72	6.14	0.1–5	0.996	8.9 ± 1.2	29.6 ± 4.1
Procyanidin C1	2.66	7.19	0.075–5	0.993	5.6 ± 0.2	18.7 ± 0.5

^1^: CV: Coefficient of variation; *R*^2^: Correlation coefficient; LOD: Limit of detection; LOQ: limit of quantitation.

**Table 3:** HPLC-DAD analysis of hydro-alcoholic extracts ^1^.

Chemical Class	Compounds	PRE	TRE	TRE Fruits	TRE Seeds	CRE1	CRE2	CRE3
Organic acids	Caftaric acid	59.40 ± 4.39	60.37 ± 1.05	51.48 ± 4.26	N.Q.	24.92 ± 1.50	74.93 ± 2.27	38.62 ± 0.73
Flavonols	Rutin	2.73 ± 0.34	N.Q.	N.Q.	N.Q.	4.32 ± 0.16	5.03 ± 0.23	5.54 ± 0.08
	Hyperoside	0.59 ± 0.02	N.Q.	N.Q.	2.40 ± 0.13	N.Q.	0.50 ± 0.01	0.95 ± 0.02
	Quercetin-3-*O*-glucoside	10.99 ± 0.51	0.52 ± 0.07	2.18 ± 0.04	4.90 ± 0.26	5.00 ± 0.19	8.05 ± 0.21	23.34 ± 0.73
	Quercetin-3-*O*-glucuronide	21.68 ± 0.91	N.D.	N.D.	N.D.	N.Q.	N.Q.	N.Q.
	Kaempferol-3-*O*-glucoside	16.81 ± 0.49	N.Q.	0.70 ± 0.10	1.43 ± 0.01	1.03 ± 0.03	0.38 ± 0.03	4.48 ± 0.04
Flavan-3-ols monomers	Catechin	N.D.	615.33 ± 42.84	N.D.	10231.06 ± 89.38	N.D.	N.D.	N.D.
	Epicatechin	N.D.	148.14 ± 13.43	N.D.	3201.16 ± 156.90	N.D.	N.D.	N.D.
	Epicatechin-3-gallate	N.D.	10.86 ± 0.34	N.D.	300.50 ± 7.11	N.D.	N.D.	N.D.
	Epigallocatechin-3-gallate	N.D.	4.94 ± 0.22	N.D.	19.45 ± 0.62	N.D.	N.D.	N.D.
Flavan-3-ols dimers and trimers	Procyanidin B1	N.D.	345.46 ± 15.43	N.D.	1690.27 ± 76.26	N.D.	N.D.	N.D.
	Procyanidin B2	N.D.	41.82 ± 2.16	N.D.	579.60 ± 51.79	N.D.	N.D.	N.D.
	Procyanidin B3	N.D.	138.93 ± 9.03	N.D.	1684.81 ± 34.85	N.D.	N.D.	N.D.
	Procyanidin C1	N.D.	14.00 ± 1.24	N.D.	703.27 ± 4.85	N.D.	N.D.	N.D.

^1^ N.D.: not detectable (under the LOD values, according to Table 2 and [18]); N.Q. not quantifiable (under the LOQ values, according to Table 2 and [18]). Results are the mean ± s.d. of at least three analysis, expressed as μg/g raisin. TRE: Turkish raisin extract; PRE: Portuguese raisin extract; CRE: commercial raisin extract.

**Table 4:** IC_50_ of the hydro-alcoholic extract from Turkish raisin for the tested biological activity.

Biological Assay	p65 Translocation	NF-κB Driven Transcription	IL-8 Promoter Activity	IL-8 Secretion
Turkey raisin fruits	>250	>250	39.5 ± 3.1	37.8 ± 2.9
Turkey raisin seeds	1.81 ± 0.182	1.34 ± 0.14	0.86 ± 0.06	0.49 ± 0.05

Results are the mean ± s.d. (μg/mL) of at least three experiments performed in duplicate (ELISA) or triplicate (transfection experiments).

## Supplementary Materials

Supplementary materials can be found at http://www.mdpi.com/1422-0067/17/7/1156/s1.

## Abbreviations

TNFα	Tumour necrosis factor alpha
NF-κB	Nuclear factor κB
IL-8	Interleukin 8
ELISA	Enzyme-linked immunosorbent assay
HPLC-DAD	High-performance liquid chromatography with Diode Array Detector
LDL	Low-density lipoprotein
IL-1β	Interleukin 1 β
ICAM-1	Intercellular Adhesion Molecule 1
GAE	Gallic acid equivalent
GA	Gallic acid
PRE	Portugal raisins extract sample
TRE	Turkey raisins extract sample
CRE1	Commercial raisins extract 1 sample
CRE2	Commercial raisins extract 2 sample
CRE3	Commercial raisins extract 3 sample
FDA	Food and Drug Administration
CV	Coefficient of variation
LOD	Limit of Detection
LOQ	Limit of Quantitation
RSD	Relative standard deviation
s.d.	Standard deviation
EGCG	Epigallocatechin-3-gallate
DMEM F12	Dulbecco’s Modified Eagle Medium F12
FCS	Foetal calf serum
TMB	3,3′,5,5′-tetramethylbenzidine
AP-1	Activator protein 1
MTT	3,4,5-dimethylthiazol-2-yl-2-5-diphenyltetrazolium bromide
