# Supplementary material for: Evaluation of the Anti-Inflammatory Activity of Raisins (Vitis vinifera L.) in Human Gastric Epithelial Cells: A Comparative Study
Source: Int J Mol Sci. 2016 Jul 19;17(7):1156. doi: 10.3390/ijms17071156 (PMC4964528; doi:10.3390/ijms17071156)
Supplement: Supplementary file 1 [file ijms-17-01156-s001.pdf]

# Supplementary Materials: Evaluation of the Anti-Inflammatory Activity of Raisins (*Vitis vinifera* L.) in Human Gastric Epithelial Cells: A Comparative Study

**Chiara Di Lorenzo, Enrico Sangiovanni, Marco Fumagalli, Elisa Colombo, Gianfranco Frigerio, Francesca Colombo, Luis Peres de Sousa, Ahmet Altindişli, Patrizia Restani and Mario Dell'Agli**

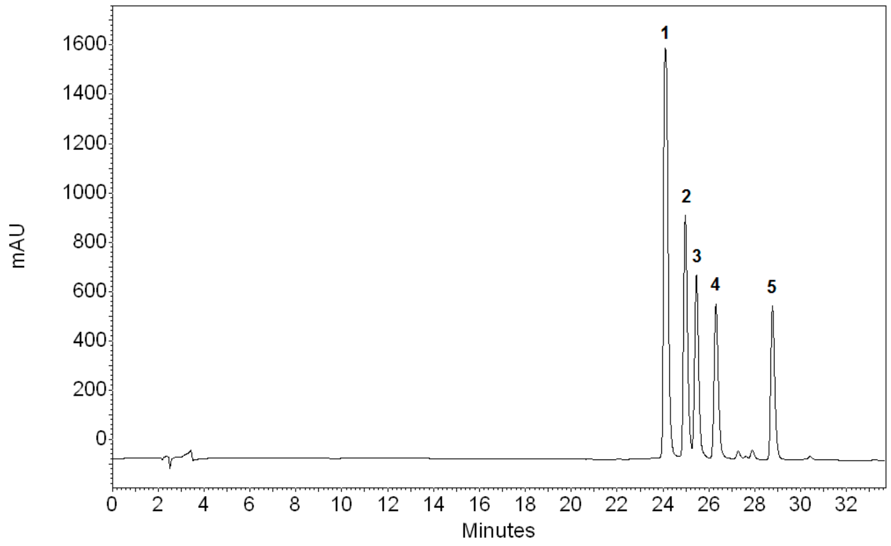


**Figure S1.** Chromatogram of a standard mixture at the concentration of 10 µg/mL revealed at 360 nm. 1: rutin; 2: hyperoside; 3: quercetin-3-*O*-glucoside; 4: quercetin-3-*O*-glucuronide; 5: kaempferol-3-*O*-glucoside.

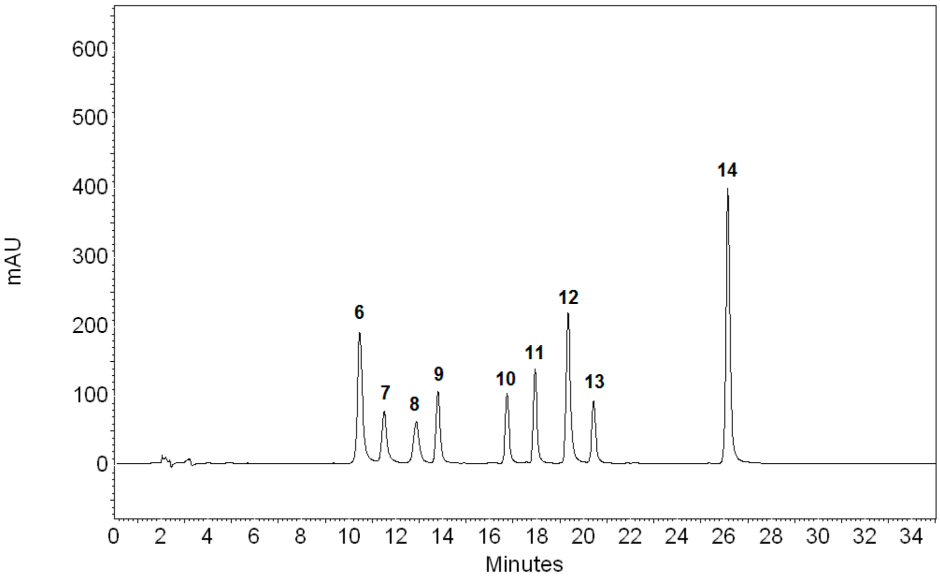


**Figure S2.** Chromatogram of a standard mixture at the concentration of 5 µg/mL revealed at 280 nm. 6: caftaric acid; 7: procyanidin B1; 8: procyanidin B3; 9: catechin; 10: procyanidin B2; 11: epicatechin; 12: epigallocatechin-3-gallate; 13: procyanidin C1; 14: epicatechin-3-gallate.

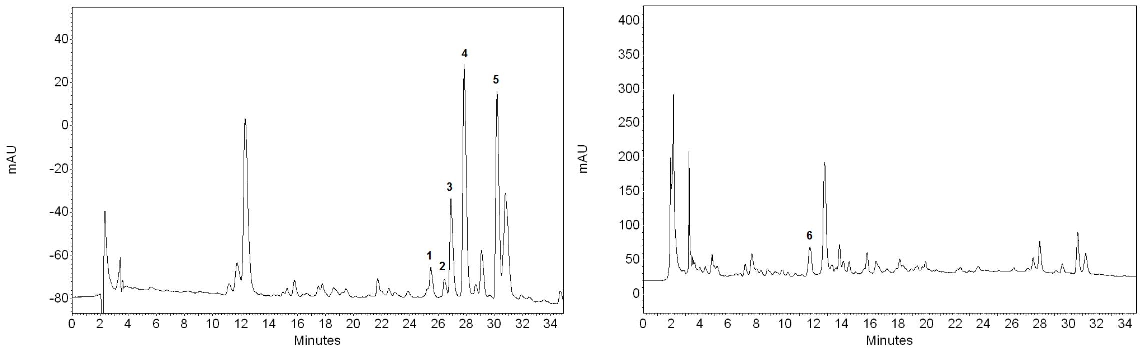


**Figure S3.** Chromatogram of sample PRE (hydroalcoholic extracts) revealed at: 360 nm (**left**); and 280 nm (**right**). 1: rutin; 2: hyperoside; 3: quercetin-3-*O*-glucoside; 4: quercetin-3-*O*-glucuronide; 5: kaempferol-3-*O*-glucoside; 6: caftaric acid.

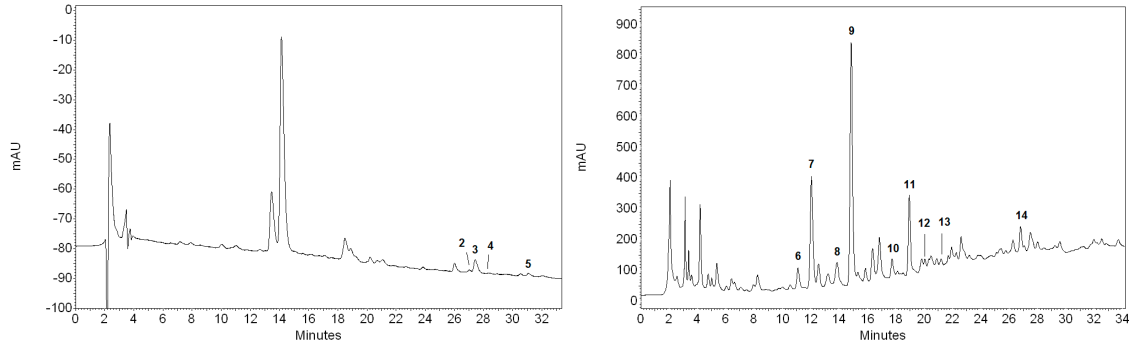


**Figure S4.** Chromatogram of sample TRE (hydroalcoholic extracts) revealed at: 360 nm (**left**); and 280 nm (**right**). 2: hyperoside; 3: quercetin-3-*O*-glucoside; 4: quercetin-3-*O*-glucuronide; 5: kaempferol-3-*O*-glucoside; 6: caftaric acid; 7: procyanidin B1; 8: procyanidin B3; 9: catechin; 10: procyanidin B2; 11: epicatechin; 12: epigallocatechin-3-gallate; 13: procyanidin C1; 14: epicatechin-3-gallate.

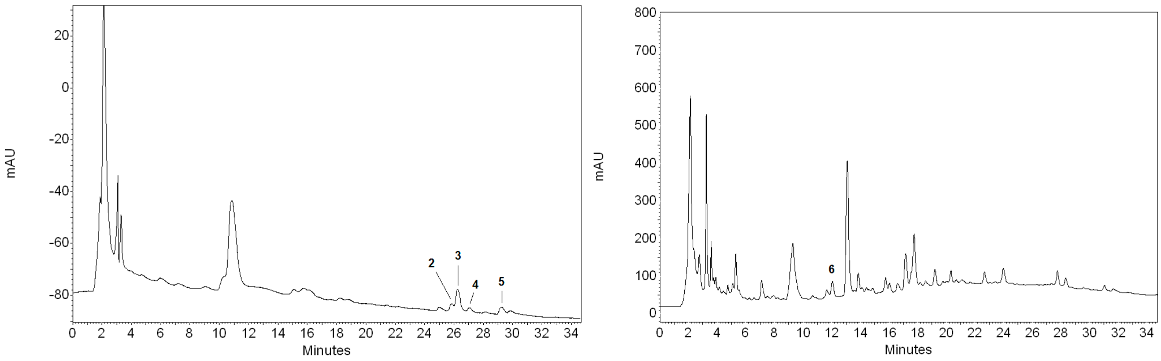


**Figure S5.** Chromatogram of sample TRE fruits (hydroalcoholic extracts) revealed at: 360 nm (**left**); and 280 nm (**right**). 2: hyperoside; 3: quercetin-3-*O*-glucoside; 4: quercetin-3-*O*-glucuronide; 5: kaempferol-3-*O*-glucoside; 6: caftaric acid.

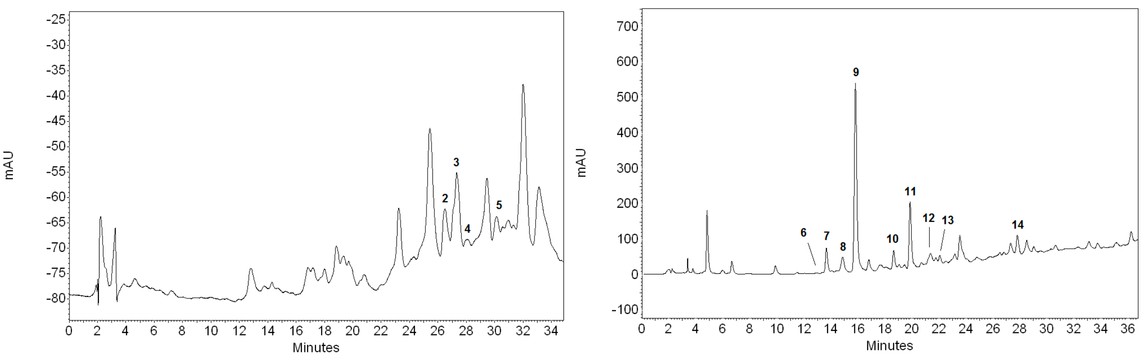


**Figure S6.** Chromatogram of sample TRE seeds (hydroalcoholic extracts) revealed at: 360 nm (**left**); and 280 nm (**right**). 2: hyperoside; 3: quercetin-3-*O*-glucoside; 4: quercetin-3-*O*-glucuronide; 5: kaempferol-3-*O*-glucoside; 6: caftaric acid; 7: procyanidin B1; 8: procyanidin B3; 9: catechin; 10: procyanidin B2; 11: epicatechin; 12: epigallocatechin-3-gallate; 13: procyanidin C1; 14: epicatechin-3-gallate.
